# Acquisition of temporal patterns from electronic health records: an application to multimorbid patients

**DOI:** 10.1186/s12911-023-02287-0

**Published:** 2023-09-19

**Authors:** Alicia Ageno, Neus Català, Marcel Pons

**Affiliations:** 1 TALP Research Center, Computer Science Department, Universitat Politècnica de Catalunya (UPC), Barcelona, Spain; 2 IDEAI-UPC Research Center, Universitat Politècnica de Catalunya (UPC), Barcelona, Spain; 3 Facultat d’Informàtica de Barcelona, Universitat Politècnica de Catalunya (UPC), Barcelona, Spain

**Keywords:** Electronic health records, Temporal data mining, Temporal association rules, Clinical decision support systems, Risk factors detection

## Abstract

**Background:**

The exponential growth of digital healthcare data is fueling the development of Knowledge Discovery in Databases (KDD). Extracting temporal relationships between medical events is essential to reveal hidden patterns that can help physicians find optimal treatments, diagnose illnesses, detect drug adverse reactions, and more. This paper presents an approach for the extraction of patient evolution patterns from electronic health records written in Catalan and/or Spanish.

**Methods:**

We propose a robust formulation for extracting Temporal Association Rules (TARs) that goes beyond simple rule extraction by considering the sequence of multiple visits. Our highly configurable algorithm leverages this formulation to extract Temporal Association Rules from sequences of medical instances. We can generate rules in the desired format, content, and temporal factors while accounting for different levels of abstraction of medical instances. To demonstrate the effectiveness of our methodology, we applied it to extract patient evolution patterns from clinical histories of multimorbid patients suffering from heart disease and stroke who visited Primary Care Centers (CAP) in Catalonia. Our main objective is to uncover complex rules with multiple temporal steps, that comprise a set of medical instances.

**Results:**

As we are working with real-world, error-prone data, we propose a process of validation of the results by expert practitioners in primary care. Despite our limited dataset, the high percentage of patterns deemed correct and relevant by the experts is promising. The insights gained from these patterns can inform preventive measures and help detect risk factors, ultimately leading to better treatments and outcomes for patients.

**Conclusion:**

Our algorithm successfully extracted a set of meaningful and relevant temporal patterns, especially for the specific type of multimorbid patients considered. These patterns were evaluated by experts and demonstrated the ability to predict risk factors that are commonly associated with certain diseases. Moreover, the average time gap between the occurrence of medical events provided critical insight into the term of these risk factors. This information holds significant value in the context of primary healthcare and preventive medicine, highlighting the potential of our method to serve as a valuable medical tool.

**Supplementary Information:**

The online version contains supplementary material available at 10.1186/s12911-023-02287-0.

## Background

The increasing adoption of Electronic Health Records (EHRs) in healthcare systems offers the potential for leveraging large amounts of health data to improve the efficiency and accuracy of medical practitioners. To support clinical diagnosis, prevention, and administrative decision-making, healthcare professionals can benefit from data mining technologies [1] and/or machine learning techniques [2]. These techniques can be used to model information from multiple data sources, such as images [3, 4], DNA sequences [5] or structured and unstructured textual data. In particular, automatic analysis of EHRs can be essential for identifying patterns, preventing errors, improving quality, reducing costs, and saving time for healthcare services. Several studies have already demonstrated the potential of data mining techniques to extract valuable insights from EHRs [6, 7].

In the context of medical diagnosis, understanding the interaction between medical instances during a patient visit is crucial for early detection and prevention of new diseases. However, research on the temporal relationships between these instances is lacking. Therefore, our goal in this work is to explore the possibility of modelling patient evolution patterns based on their clinical histories, to identify interesting associations such as drug side effects, recurrent chronic symptoms, and co-occurring diagnoses. By doing so, we hope to improve the medical tools available to physicians and ultimately provide more personalized patient care.

EHRs are essential sources of temporal information that provide a comprehensive understanding of healthcare events. To extract implicit, non-trivial, and potentially useful abstract information from large collections of temporal data, temporal data mining techniques have been explored [8]. Our approach focuses on one of these techniques, namely temporal association rules [9–16]. We aim to develop a new variant of temporal association rule that incorporates our formulae for computing the associated measures of support and confidence. Furthermore, we will propose two variants of a new algorithm for mining these temporal association rules.

Although our framework will be general, in this article we will focus on its application to knowledge discovery for risk prediction and disease diagnosis in multimorbid patients. These patients are often affected by multiple disorders and symptomatologies, making them a prevalent issue in certain clinical contexts, such as primary care. Despite their prevalence, the treatment of multimorbid patients has not been extensively studied. To address this research gap, we aim to develop a robust framework for extracting temporal association rules from multi-attribute data.

Temporal association rules are a type of sequential pattern mining that aims to identify associations between events that occur in a particular temporal order. By analyzing the timing and sequence of events, temporal association rules can provide valuable insights into the underlying relationships between different health conditions or symptoms. Our approach will focus on the temporal aspect of the data, which will be used as an input parameter and obtained as part of the results. We will also incorporate the ability to express healthcare instances at different levels of abstraction, allowing us to extract patterns with different degrees of generalization^1^. Our ultimate goal is to develop a comprehensive framework for extracting these rules from multi-attribute data in order to improve our understanding of the complex relationships between health conditions.

Association rule mining is a data mining technique that aims to discover relationships, patterns, or rules among variables in a dataset. The origins of association rule mining can be traced back to the Apriori algorithm [17]. Since then, several extensions have been developed for different purposes [18], such as sequential pattern mining [19] which aims to identify patterns of ordered events in a dataset by finding frequent sequences of itemsets. An example of such an extension is the Generalized Sequential Pattern (GSP) algorithm [20]. The problem of learning rules from time series data, where the antecedent tends to appear in conjunction with the consequent according to certain time constraints, is called *temporal association rule mining*.

A temporal association rule (TAR) is a type of temporal relationship that describes a connection between an antecedent and a consequent. Although temporal pattern mining in general (and temporal association rule mining in particular) has received considerable attention in recent years, one of the main problems in this area is the lack of visibility of most of the work, as there is no standard terminology to refer to. Different formulations of TARs can be found in the literature, each with different approaches to computing support and confidence, and each introducing the temporal component in different ways, making it difficult to find and compare proposals and studies in this area.

Mooney and Roddick [21] describes several sequential pattern mining approaches, including temporal sequences and more specifically TARs. These approaches extract patterns with only one antecedent and one consequent. Eventually, the antecedent may consist of several sequential elements, but there is no way to detail temporal constraints among them. However, our algorithm has been designed to extract rules with different numbers of temporal steps (allowing a different interval [t1, t2] for each step), attributes and levels of concept abstraction.

Furthermore, a comprehensive overview of the different methods for temporal association rule mining is provided in [9]. The work proposes a taxonomy in which they classify the existing approaches at a first level into two categories, based on the use of the time variable: (1) “time as implied component”, those that use temporally ordered datasets to discover temporal constraints, and (2) “time as integral component” those that integrate the time variable as another attribute of the data and analyze the temporal aspects in which the rules occur. According to their definition, our methodology would be a hybrid belonging to both first-level categories, “time as implied” (with second-level category “sequential”) and “time as integral component” (with second-level category “time-interval”). This is so because, on the one hand, the time variable provides order and temporal constraints to the EHR database, and on the other hand it is also used as a parameter in the process of pattern extraction (in the form of time gaps). The article also describes a large number of applications in the areas of medicine and healthcare (such as [22–25]). However, none of them is directly comparable to our approach, as they only belong to one of the categories in the taxonomy.

In [10] and [11], an algorithm for the extraction of temporal association rules is presented, which deals with complex temporal patterns, represented through the formalism of Temporal Abstractions. This algorithm allows dealing both with events with a certain time duration and with events that occur at a single moment in time.

In their paper, Zhan et al. [12] present a more generalized form of temporal association rules. These extended temporal association rules take the form of an implication $X_1 \overset{t_1}{\Rightarrow }\ X_2 \overset{t_2}{\Rightarrow }\ ... \overset{t_{p-1}}{\Rightarrow } X_p$ with $p \ge 2$, where each $t_i$ is a time constraint. In addition, a modified formulation for computing support and confidence for these rules is defined. Furthermore, the authors introduce another form of temporal association rules, $X_1 \overset{\scriptscriptstyle T_{1}}{\Rightarrow } X_2 \overset{\scriptscriptstyle {T_2}}{\Rightarrow } ... \overset{\scriptscriptstyle {T_{p-1}}}{\Rightarrow } X_p$, where $T_i=\big [t_{i_1}, t_{i_2}\big ]$ are time intervals with $t_{i_1}<t_{i_2}$. They use these definitions to propose a fast algorithm for mining temporal association rules, which they test on both synthetic and real datasets, demonstrating excellent performance. Our work is based on a combination of ideas from [10] and [12]. By leveraging these insights, we aim to advance the application of temporal association rules and develop more effective methods for knowledge discovery.

## Methods

### Electronic health record (EHR) dataset

Our dataset contains information on patient visits at Primary Care Centers (CAP) in Catalonia. This dataset has been provided by IDIAP JGol [26] and consists of EHRs of multimorbid patients visited between 2010 and 2016. In our case, a multimorbid patient is a patient whose medical history includes at least one of the following symptoms, which must have occurred when the patient was over 50 years of age:

- Transient ischaemic attack
- Haemorrhagic strokes
- Ischaemic strokes
- Other types of strokes
- Stroke-related sequelae
- Lung cancer
- Colorectal cancer
- Acute myocardial infarction
- Exitus

**Fig. 1:**
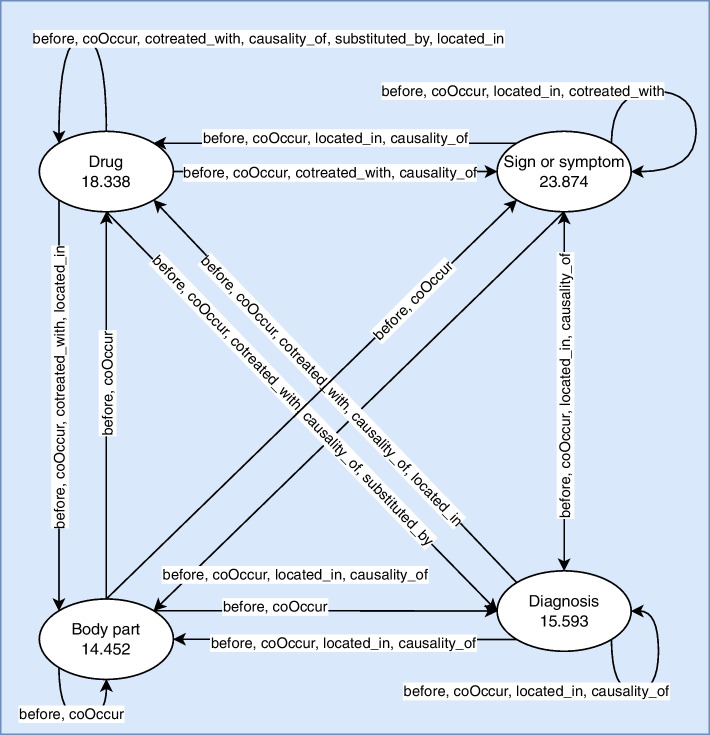
Data model of IDIAP dataset

Our EHRs, written in Catalan and/or Spanish, are composed of four sections: reason for the consultation, medical examination, evaluation and medical treatment plan. These EHRs were manually annotated and represented in a graph format (Fig. 1) with four types of nodes: *Body part*, *Diagnosis*, *Drug*, and *Sign or symptom*. These nodes will represent our healthcare instances. In addition, six types of relationship were annotated: *before*, *causality_of*, *coOccur*, *cotreated_with*, *located_in*, and *substituted_by*. The “Appendix” contains a detailed description of node properties (or attributes) and relationships. As the corpus has been manually annotated, it may contain some inconsistencies. Indeed, the manual generation of high quality annotated medical records for such complex concepts and relationships has been particularly challenging, highlighting the need for further development of resources for annotating EHRs.

The IDIAP dataset contains information about 320 patients making a total of 72,257 healthcare instances, distributed as shown in Table 1. We focus on two node attributes, the *code* associated with each node and the *raw_text*, which is the description that the practitioner entered for each medical instance. The table shows the number of different codes, the number of different text descriptions, and the total number of instances for each type of node. There are a total of 14,330 visits. Each patient attended an average of 44.78 visits during these six years, with a standard deviation of 34.83. The patient with the most visits had 205 visits and the patient with the fewest visits had only 1 visit. Visits have an average of 5.04 healthcare instances. The mean time between two consecutive visits for a patient is 42.83 days and the standard deviation is 86.29 days, with a minimum of 1 day and a maximum of 2,005 days (5.5 years). These annotated EHRs have been imported into a Neo4J [27] database, from which the information can be extracted.

**Table 1:** Distribution of healthcare instances and their attributes before preprocessing

Node type	Codes	Raw_texts	Instances
Body part	601	5184	14452
Diagnosis	1193	6429	15593
Drug	796	3742	18338
Sign or symptom	249	10455	23874
Total	2839	25810	72257

### Data preparation

The first stage of data preprocessing consisted of the filtering of the annotations according to the *time* and *certainty* attributes (see “Appendix”). The following preprocessing steps were applied to the *raw_text* attribute: case folding, stopword removal and string distance. The latter means that for those *raw_text* values that have more than three characters, we grouped all the values that had a Levenshtein distance equal to or less than two. Furthermore, some mistyped codes were corrected.

Table 2 shows the number of different values for the attributes *code* and *raw_text* after preprocessing. Despite this preprocessing, we can see that our dataset is quite limited. The number of different healthcare instances is still large with respect to the number of visits, so the frequency of occurrence of each medical instance is small. This may imply low supports for the associations, which we will try to increase through generalization.

**Table 2:** Distribution of healthcare instances and their attributes after preprocessing

Node type	Codes	Raw_texts	Instances
Body part	601	4103	14452
Diagnosis	1158	4003	15012
Drug	792	1930	18086
Sign or symptom	225	8138	22835
Total	2776	18174	70385

Different levels of generalization can be applied to each type of *code*, except for the *Body part* nodes (see “Appendix” for a description of the levels). The use of *raw_text* was discarded because the small size of the dataset did not allow sufficient generalization. Table 2 shows the high variability of the raw texts compared to the number of occurrences. In this work, we have decided to use a level of generalization where we use level 2 for *Diagnosis*, level 5 for *Drug* and level 2 for *Sign or symptom*. We consider that these levels provide a good compromise between interpretability and generalization. When we refer to a code level, we specify the maximum code level allowed. However, if a given node does not provide the specified level, its maximum level of generalization will be used.

### Temporal association rules formulation

In this section, we will present the formulation for computing our temporal association rules. As mentioned above, this formulation is an extension of the one defined in [12].

Let $\mathcal {J}$ be a set of healthcare instances, denoted by $I_1, I_2, \dots , I_m$. Let $\mathcal {D}$ be a set of temporal transactions, where each transaction $(S_i, t_i)$ corresponds to a healthcare visit $S_i$ attended at time $t_i$. First, we define temporal association rules of the form *X* $\overset{t}{\Rightarrow }\ Y$, where *X* $\mathcal {\subset J}$, *Y* $\mathcal { \subset J}$ and *t* is a unit of time (in our context, days). These rules imply that a time gap of *t* has elapsed between an occurrence of instances *X* and an occurrence of instances *Y*. Unlike conventional association rules, *X* $\mathcal {\cap }$ *Y* need not necessarily be $\emptyset$, since it makes sense for the same healthcare events to continue to occur after some time.

Let

$$\begin{aligned}{} & {} g_{S_{i}}^{p} : \mathcal {P}(\mathcal {J}) \rightarrow \{0,1\}\\{} & {} g_{S_{i}}^{p}(X) =\left\{ \begin{array}{ll} 1 &{} \text {if } S_{i} \text { exists and contains } X \text { for patient } p\\ 0 &{} \text {else } \end{array}\right. \end{aligned}$$

where $S_{i}$ is a visit at time (day) *i*. Then

$$\begin{aligned}{} & {} g_{S_{i},t}^{p} : \mathcal {P}(\mathcal {J})^{2} \rightarrow \{0,1\} \\{} & {} g_{S_{i},t}^{p}(X, Y) =\min \left( g_{S_{i}}^{p}(X), g_{S_{i+t}}^{p}(Y)\right) \end{aligned}$$

where *p* is a patient, $S_{i}$ is a visit at time *i*, and *t* is a time interval (or temporal gap) measured in days. This function returns a value of 1 if, at time *i*, the patient *p* has healthcare instances *X*, and at time $i+t$, the same patient *p* has instances *Y*. Otherwise, the function returns a value of 0. In our formulation, the parameter $\emptyset$ simply indicates the occurrence of a visit at the given time. For example, $g_{S_{i},t}^{p}(X, \emptyset )$ is equal to 1 if, for a given patient *p*, $S_{i}$ exists and contains *X*, and there is a visit $S_{i+t}$.

Let

$$\begin{aligned} h_{t}: \mathcal {P}(\mathcal {J})^{2} \rightarrow \mathbb {N}, \;\;\;\; h_{t}(X, Y)=\sum _{p} \sum _{S_{i} \in D} g_{S_{i},t}^{p}(X, Y) \end{aligned}$$

$h_{t}$ is the count support for *X*, *Y*. The inner sum accounts for the number of times the patient *p* presents *X* at time *i* and *Y* at time $i+t$. The outer sum is done to take into account all the patients. This formula is used to compute the support and confidence of temporal association rules *X* $\overset{t}{\Rightarrow }\ Y$.

Furthermore, we extend these temporal association rules to deal with time intervals. These rules are expressed as ${X \overset{T}{\Rightarrow }\ Y}$, where ${T=\left[ t_{1},t_{2}\right] \left( t_{1}<t_{2}\right) }$. We denote $t_{1}$ as $min\_gap$ and $t_{2}$ as $max\_gap$. Then:

$$\begin{aligned}{} & {} h_{T}: \mathcal {P}(\mathcal {J})^{2} \rightarrow \mathbb {N} \\{} & {} h_{T}(X, Y)=\sum _{p} \sum _{S_{i} \in D} \min \left( \sum _{t \in T} g_{S_{i},t}^{p}(X, Y), \;\; 1\right) \end{aligned}$$

$h_{T}$ is the count support for *X*, *Y*. The inner sum accounts for the number of times that a patient *p* presents *X* at time *i* and *Y* at time $i+t$ in a set of visits $S_{i}$. The *min* function ensures that, in case the association $X \overset{T}{\Rightarrow }\ Y$ exists, it is counted only once for each consequent. The summation $\sum _{S_i \in D}$ takes into account all visits of a patient, while the outer summation accounts for all patients. Using these formulae, we can define our version of the following measures:

1 $$\begin{aligned}{} & {} support\ (X \overset{T}{\Rightarrow }\ Y)=\dfrac{h_{T}(X, Y)}{h_{T}(\emptyset , \emptyset )}\end{aligned}$$

2 $$\begin{aligned}{} & {} confidence\ (X \overset{T}{\Rightarrow }\ Y)=\dfrac{h_{T}(X, Y)}{h_{T}(X, \emptyset )}\end{aligned}$$

3 $$\begin{aligned}{} & {} rulesRespectConsequent\ (X \overset{T}{\Rightarrow }\ Y)=\dfrac{h_{T}(X, Y)}{h_{T}(\emptyset , Y)}\end{aligned}$$

4 $$\begin{aligned}{} & {} lift\ (X \overset{T}{\Rightarrow }\ Y)=\dfrac{support(X, Y)}{support(X, \emptyset )\cdot support(\emptyset , Y)}\end{aligned}$$

5 $$\begin{aligned}{} & {} leverage\ (X \overset{T}{\Rightarrow }\ Y) = support(X, Y) \nonumber \\{} & {} \qquad \qquad \qquad \qquad - support(X, \emptyset )\cdot support(\emptyset , Y) \end{aligned}$$

In particular, *rulesRespectConsequent* (Eq. 3) is a new measure requested by the experts during the evaluation phase (see “Results” section). It denotes the number of medical cases that feature the antecedents in the elapsed gap, out of all the cases that present the consequent. The rest are widely known measures.

When applying the previous formulation to automatically infer patterns that help to understand the clinical conditions of multimorbid patients, we faced a dilemma. On the one hand, temporal association rules could reflect recurrent patterns presented by the patients, extracted as the repetition of medical events frequently presented by any patient. The rationale of this implementation is consistent with the conventional Apriori algorithm, which does not distinguish the agent presenting the patterns, so this approach will be referred to as apriori-like. On the other hand, temporal association rules may reflect patterns that frequently occur among patients, ignoring the repetition of healthcare events within each patient. This approach aims to generate common rules that are present in the clinical data and might be more useful for providing information about conventional (non-multimorbid) patients, so it will be referred to as patient-oriented. The previous formulation needs to be adapted to these two approaches, though it is not included here to make the text more readable.

The formulations presented here can be extended to cases where a sequence of antecedents or temporal steps leads to a given consequent. The resulting temporal association rules have the form $X_1 \overset{T_1}{\Rightarrow }\ X_2 \overset{T_2}{\Rightarrow }\ \cdots \overset{T_n}{\Rightarrow }\ X_n$, where each *X* represents either a single instance of a diagnosis, drug, body part, symptom or sign or a combination of these. In this notation, the arrows represent a time interval between each sequence of events, or different healthcare visits occurring at different times. These temporal steps can be time specific (*t*) or intervals (*T*).

The resulting formulation is a modification of the one presented in [12]. Let $t=(t_{1},\cdots , t_{q-1})$

$$\begin{aligned}{} & {} g_{S_{i},t}^{p}: \mathcal {P}(\mathcal {J})^{q} \rightarrow \{0,1\} \\{} & {} g_{S_{i},t}^{p}(X_{1}, X_{2},..., X_{q})=\\{} & {} \qquad min \left( g_{S_{i}}^{p}(X_{1}), g_{S_{i+t_{1}}}^{p}(X_{2}),...,g_{S_{i+t_{1}+...+t_{q-1}}}^{p}(X_{q})\right) \end{aligned}$$

The computation of the support count function changes according to the version. For the apriori-like approach:

6 $$\begin{aligned}{} & {} h_{t}^{p}: \mathcal {P}(\mathcal {J})^{q} \rightarrow \mathbb {N}\nonumber \\{} & {} h_{t}^{p}(X_{1}, X_{2},..., X_{q})=\sum _{S_{i} \in D} g_{S_{i},t}^{p}(X_{1}, X_{2},..., X_{q}) \end{aligned}$$

And for the patient-oriented approach:

7 $$\begin{aligned}{} & {} h_{t}^{p}: \mathcal {P}(\mathcal {J})^{q} \rightarrow \mathbb {N}\nonumber \\{} & {} h_{t}^{p}(X_{1}, X_{2},..., X_{q})=min\left( \sum _{S_{i} \in D} g_{S_{i},t}^{p}(X_{1}, X_{2},..., X_{q}), 1\right) \end{aligned}$$

Summing up for all the patients:

8 $$\begin{aligned} h_{t}(X_{1}, X_{2},..., X_{q})=\sum _{P} h_{t}^{p}(X_{1}, X_{2},..., X_{q}) \end{aligned}$$

Then, the rule indicators can be computed with the same formulae for both versions:

$$\begin{aligned}{} & {} {{support}}(X_{1} \overset{t_{1}}{\Rightarrow } X_{2}... \overset{t_{q-1}}{\Rightarrow } X_{q})=\dfrac{h_{t}(X_{1}, X_{2},..., X_{q} )}{h_{t}(\emptyset ,...,\emptyset )}\\{} & {} {{confidence}}(X_{1} \overset{t_{1}}{\Rightarrow } X_{2}... \overset{t_{q-1}}{\Rightarrow } X_{q})= \dfrac{h_{t}(X_{1}, X_{2},..., X_{q})}{h_{t}(X_{1}, X_{2},..., X_{q-1}, \emptyset )}\\{} & {} {{rulesRespectConsequent}}(X_{1} \overset{t_{1}}{\Rightarrow } X_{2}... \overset{t_{q-1}}{\Rightarrow } X_{q})= \dfrac{h_{t}(X_{1}, X_{2},..., X_{q})}{ h_{t}(\emptyset , ..., X_{q})}\\{} & {} {{lift}}(X_{1} \overset{t_{1}}{\Rightarrow } X_{2}... \overset{t_{q-1}}{\Rightarrow } X_{q})= \dfrac{h_{t}(X_{1}, X_{2},..., X_{q})}{ h_{t}(X_{1}, X_{2},..., X_{q-1}, \emptyset )*h_{t}(\emptyset ,..., X_{q})}\\{} & {} {{leverage}}(X_{1} \overset{t_{1}}{\Rightarrow } X_{2}... \overset{t_{q-1}}{\Rightarrow } X_{q})= h_{t}(X_{1}, X_{2},..., X_{q})\\{} & {} \qquad \qquad \qquad \qquad \qquad \qquad \qquad \qquad \quad - h_{t}(X_{1}, X_{2},..., X_{q-1}, \emptyset ) * h_{t}(\emptyset ,..., X_{q}) \end{aligned}$$

The previous formulation is also extended to deal with time intervals, although we will not go into the details here.

### Temporal association rules extraction

Using the previous formulation, we have developed two algorithms for mining temporal association rules using both apriori-like and patient-oriented approaches. These algorithms are based on the work of  [10] and  [12]. Both algorithms leverage pre-computed data structures based on the generalization levels of healthcare instances. These algorithms also take as input the minimum support and confidence required (*minsup* and *minconf*) and a list of *n* time intervals $[T_1, T_2 \cdots T_n]$ whose length represents the maximum number of temporal steps to be considered for the rules.

**Fig. 2:**
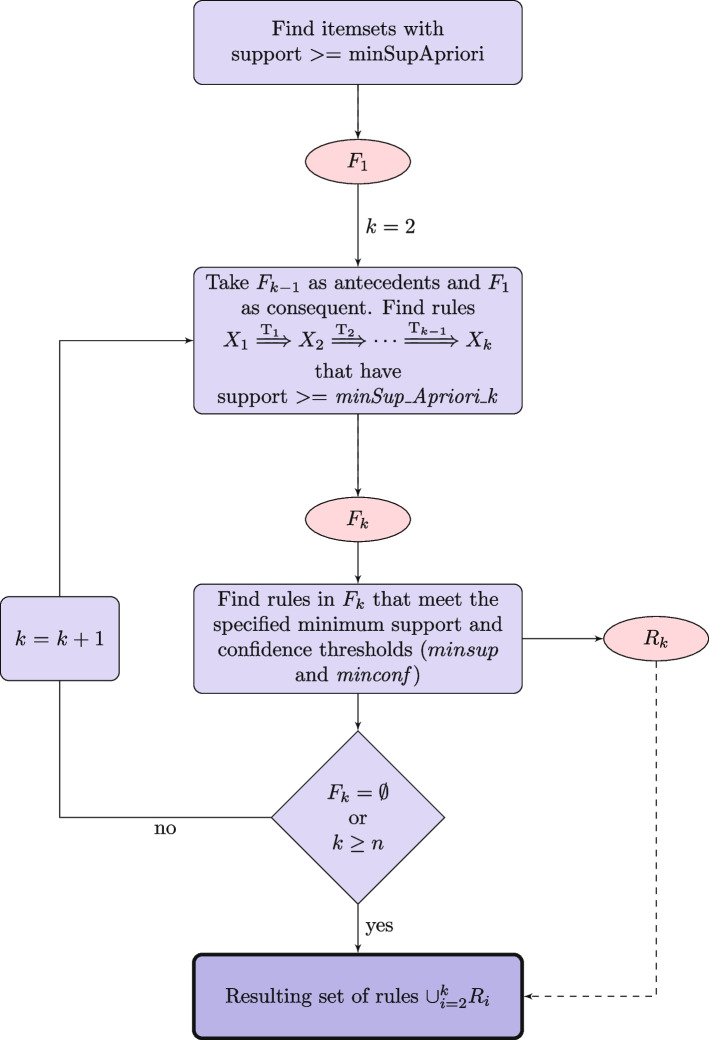
Flow diagram of the temporal association rule mining algorithm. Pink ovals represent intermediate item sets ($F_1$) and sets of rules ($F_k$ and $R_k$)

Both algorithms share a common structure, but the main difference between them lies in the counting methodology. The apriori-like approach counts all consecutive events that satisfy temporal constraints, while the patient-oriented approach counts patients that satisfy temporal constraints across their visits. In particular, if the same set of consecutive events satisfies the condition $a_i \overset{T_1}{\Rightarrow }\ a_j$ in one patient multiple times, it is counted only once. The differences between the two algorithms are implemented in the code by calculating support and confidence differently, as described in the formulations. In addition, a new variable, *minSup_Apriori*, is introduced to take into account the minimum support of the previous time steps. This variable is a percentage value. Figure 2 provides a brief description of the steps involved in the algorithm.

The algorithms return a list of rules along with their corresponding metrics: *support*, *confidence*, *lift*, *leverage*, and *rulesRespectConsequent*. Furthermore, the average time gap between each successive event of the rules is computed. For apriori-like, the average time gap is calculated by taking the average of all the time gaps that meet the thresholds for the specific step. For patient-oriented, it is calculated by taking the average of a list, where each item is the average of the time gaps satisfying the thresholds presented by a patient.

By providing all these parameters, we aimed to design a highly adaptable code, not only in terms of *support* and *confidence* measures but also to allow the extraction of rules with different numbers of temporal steps, different time gaps and different levels of abstraction for each instance. The aim is to provide a powerful tool for generating rule sets that can be applied across various domains and applications. The source code of the algorithms is available at [28].

## Results and discussion

A good diagnosis must take into account the patient’s medical history. As mentioned in the “Background” section, we have tested our framework mainly to obtain patterns to help in diagnosis or to detect risk factors that may somehow derive certain diseases. Since there is no gold standard of valid patterns, the evaluation of the obtained results in clinical practice is usually based on the interpretation by domain experts, who may consider various criteria to assess their quality, relevance, and clinical significance. It’s important to note that these criteria may vary depending on the specific clinical context, disease area, or research objectives.

In our work, the evaluation of the generated patterns was carried out by three experts, experienced primary care physicians from IDIAP [26]. As the usefulness of a pattern ultimately depends on the experts’ ability to apply it or integrate it into the decision-making process, we conducted initial test evaluations using a small set of patterns. After the preliminary evaluation process, the authors and the experts agreed on two specific criteria:

- Correctness: Experts assess whether the patterns align with known clinical knowledge, established medical guidelines, or domain expertise. Patterns are intuitive and logically consistent. Experts consider whether the patterns are easy to understand and provide clear insights into patient characteristics, disease trajectories, risk factors, treatment effectiveness or other relevant clinical factors. They also assess whether the patterns can effectively predict future events, disease progression, treatment response, or adverse outcomes.
- Relevance: Experts assess whether the patterns reflect meaningful associations or dependencies that have practical utility for patient care, disease progression, treatment response or other clinical outcomes. They assess whether the patterns can contribute to improved diagnosis, prognosis, treatment selection, risk assessment or patient monitoring. Therefore, experts may consider a pattern that does not represent known medical knowledge to be relevant.

We then worked together to develop a set of labels to assess both criteria. These labels allow us to determine the level of agreement among experts on our findings. The labels for *Correctness* evaluation can be defined as follows:

- **Totally incorrect:** The rule is completely wrong or contradicts medical knowledge.
- **Totally correct:** The rule is completely right and aligns with medical knowledge.
- **Partially correct (temporal facet):** The rule is correct, but any of the temporal gaps between antecedents and consequent are not the expected ones.
- **Partially correct (clinical facet):** The rule is partially correct due to the absence or excess of any antecedent.
- **Partially correct (both facets):** The rule is partially correct due to the absence or excess of any antecedent and the inappropriateness of any of the time gaps.

In terms of *Relevance*, the rules have been sorted according to the following classes:

- **Not relevant:** The rule is considered worthless (it presents no significant clinical relevance).
- **Relevant and known:** The rule has significant clinical relevance, and it is a well-known implication (common knowledge among experts).
- **Relevant and unknown:** The rule has significant clinical relevance and should be studied further, as it is not a well-known implication.

It is important to emphasize that we are not looking for strict cause-effect relationships here but for patterns of behaviour. This means that there can be any kind of relationship between antecedents and consequent.

We generated several packs of rules, each representing different parameterizations. Due to the large number of combinations of parameter values, we fixed some of them and focused on testing both approaches (apriori-like and patient-oriented) under the following time constraints, which were agreed upon by the experts. For simplicity, the same time constraints were applied to each of the several possible temporal steps of the rules:

- Shortest-term rules: medical events that take place within 1 to 10 days (39% of the visits in our test set occurred within this interval).
- Short-term rules: medical events that occur within 5 to 30 days (45% of the visits).
- Mid-term rules: medical events that take place within 20 to 90 days (33% of the visits).
- Long-term rules: medical events that occur within 60 to 120 days (11% of the visits).

These packs of rules were individually annotated by the experts using the previous labels. Then they came to a common agreement. The experts have emphasized that the relevance of the patterns depends heavily on the time gaps. It is very different to be diagnosed with a cold than it is to be diagnosed with a malignant neoplasm. If a patient has a cold, it may be relevant to know what happened 3-15 days before, but in the case of malignant neoplasm, we would like to consider what happened, for instance, in the last 30-300 days.

Nevertheless, these executions still produce hundreds of rules, depending on the values of the parameters *min_sup* and *min_conf*. To filter these rules for each *min_gap* - *max_gap* parameterization, the first selection criterion was that they had to contain more than one temporal step, since having multiple temporal steps is essential for testing our methodology. These resulting multi-step rules were sorted according to *lift* and then *rulesRespectConsequent* values, and the top 20 were selected^2^. *Lift* measures the ratio of the confidence of the rule to the expected confidence of the rule (Eq. (4,5)). Overall, *lift* quantifies the degree of association between the elements on the antecedents and the consequent of a rule, and higher values of lift indicate stronger association. In this work, the sorting criterion for the rules was chosen on the basis of the lift, and in the case of ties, the frequency of occurrence would prevail.

The minimum support for each pack was adjusted based on the chosen approach and time gap. Initially, we started with values around $5\%$ and gradually decreased them to obtain rules with more temporal steps. For instance, the minimum *minSup* was set to $0.5\%$ to generate 3-step rules for the shortest-term time gap of the apriori-like approach. As for *minConf*, it was set at a high level with a minimum value of $80\%$.

After each pack was independently annotated by the three experts, we assessed the agreement achieved. Since the classification categories are not ordinal and three annotators were involved, we decided to use Fleiss’ Kappa measure [29]. The results obtained (see Table 3), highlight the subjectivity and difficulty of the evaluation process. According to the standard interpretation [30], positive values below 0.2 would be considered as a “slight agreement”. However, we would have expected a higher (at least “fair”) agreement, that would have indicated that these patterns corresponded to common medical knowledge among physicians. There may be two reasons for these figures. On the one hand, the small volume of data might produce patterns that reproduce less frequent phenomena (uncommon behaviour in the experts’ knowledge), which may justify the inconsistency among the experts. On the other hand, the experts complained that the codes were too general, which made the evaluation harder. They also pointed out that, in many cases, healthcare instances are findings that you discover when performing other examinations. This is coherent with our interpretation of our rules as patterns of behaviour rather than strict cause-effect relationships.

**Table 3:** Fleiss’ Kappa agreement among experts

Approach	Correctness	Relevance
apriori-like	0.105	0.038
patient-oriented	0.119	0.002

Tables 4 and 5 show respectively a summary of the percentages of *Correctness* and *Relevance* in the annotated packs after the expert consensus. The results depend strongly on the approach. For apriori-like, regarding *Correctness*, we can see that two-thirds of the patterns are considered correct, indicating that our algorithm is extracting sound data. However, the patient-oriented approach performs worse. One explanation for this positive tendency towards the apriori-like method stems from the dataset used for the pattern extraction, which consisted of multimorbid patients whose visits present recurrent medical conditions and are therefore more suitable for apriori-like characteristics. The difference between the approaches is particularly relevant for long-term patterns, probably because the shortage of data is more critical when capturing long-term phenomena.

**Table 4:** Summary of *Correctness* results after consensus

	Correctness				
	Incorrect	Correct	Par-Temp	Par-Clinic	Par-Both
apriori-like					
Shortest-term	25	65	10	0	0
Short-term	35	60	5	0	0
Mid-term	50	45	5	0	0
Long-term	10	80	10	0	0
Global	30	62.5	7.5	0	0
patient-oriented					
Shortest-term	50	45	0	5	0
Short-term	45	35	10	10	0
Mid-term	45	35	10	10	0
Long-term	43.3	23.3	16.6	10	6.6
Global	45.82	34.57	9.15	8.75	1.65

As to *Relevance* consensus, the percentage of relevant known rules is greater for apriori-like. Once more, it seems that this approach works better at finding well-known associations in this particular dataset. However, the “Relevant and unknown” category seems to be an issue: there are no patterns at all. Prior to consensus, a few cases were annotated by the experts with this category, but it did not appear anymore after agreement. This is unfortunate, since we considered it a very interesting tag. We hypothesize that this happened because the experts found it difficult to annotate cases as relevant, especially those that were frequent but did not conform to conventional patterns.

**Table 5:** Summary of *Relevance* results after consensus

	Relevance		
	No-Rel	Rel-Known	Rel-Unknown
apriori-like			
Shortest-term	35	65	0
Short-term	70	30	0
Mid-term	70	30	0
Long-term	10	90	0
Global	46.25	53.75	0
patient-oriented			
Shortest-term	50	50	0
Short-term	60	40	0
Mid-term	45	55	0
Long-term	63.3	36.6	0
Global	64.57	35.4	0

Table 6 shows the relationship between correctness and relevance. As expected, most of the cases are either totally correct and relevant-known or incorrect and non-relevant.

**Table 6:** Correctness versus relevance percentages

	Relevance	
Correctness	Not Relevant	Relevant
*apriori-like*		
Incorrect	30	3
Correct	21	39
Par-Temporal	0	7
Par-Clinical	0	0
Par-Both	0	0
*patient-oriented*		
Incorrect	44.5	1
Correct	0	33
Par-Temporal	4.5	6
Par-Clinical	4.5	4.5
Par-Both	2	0

Tables 7 and 8, present, for each approach, an example of a correct and relevant rule per time gap, as agreed by the three experts. As mentioned above, the average time gap value is expressed in days^3^. For the sake of clarity, healthcare instances have been represented using the textual descriptions of their codes.

**Table 7:** Apriori-like’s correct and relevant pattern samples, grouped by time constraint type

Respiratory symptom/complaint other $\overset{5}{\Longrightarrow }$ Cough $\overset{6}{\Longrightarrow }$ Respiratory symptom/complaint other $\overset{6}{\Longrightarrow }$ Respiratory symptom/complaint other $\overset{4}{\Longrightarrow }$ Throat symptom/complaint
Respiratory symptom/complaint other $\overset{16}{\Longrightarrow }$ Cough $\overset{16}{\Longrightarrow }$ Respiratory symptom/complaint other, Acute nasopharyngitis [common cold]
Entire limb $\overset{47}{\Longrightarrow }$ Musculoskeletal sympt/complt other $\overset{45}{\Longrightarrow }$ Pain general/multiple sites
Entire thorax $\overset{96}{\Longrightarrow }$ Angina pectoris $\overset{88}{\Longrightarrow }$ Pain respiratory system, Entire thorax

**Table 8:** Patient-oriented’s correct and relevant pattern samples, grouped by time constraint type

Shortness of breath/dyspnoea $\overset{5}{\Longrightarrow }$ Fever $\overset{5}{\Longrightarrow }$ Paracetamol
Respiratory symptom/complaint other $\overset{16}{\Longrightarrow }$ Pain general/multiple sites $\overset{16}{\Longrightarrow }$ Cardiovascular sympt/complt other $\overset{16}{\Longrightarrow }$ Shortness of breath/dyspnoea
Respiratory symptom/complaint other $\overset{43}{\Longrightarrow }$ Paracetamol $\overset{45}{\Longrightarrow }$ Skin symptom/complaint other
Psychological symptom/complt other $\overset{79}{\Longrightarrow }$ Pain general/multiple sites $\overset{87}{\Longrightarrow }$ Musculoskeletal sympt/complt other, Neurological symptom/complt. other

As shown in Table 7, the first two patterns are examples of combinations of typical short-term symptoms associated with respiratory disorders, especially at certain times of the year. The third pattern is an example of a very general symptom combined with a body part that provides specific information about the location of the symptom, in this case indicating a chronic osteoarticular pain such as arthrosis. The last pattern is an example of the long-term development of multi-pathology patients with severe chronic cardiac and respiratory pathologies.

Regarding Table 8, the first pattern is an example of a short-term respiratory infection context, which includes fever and is treated with paracetamol. The second pattern shows a combination of symptoms from patients with both respiratory and cardiac conditions. The third pattern could be an example of a possible mid-term side effect, where a respiratory infection treated with paracetamol results in symptoms in the skin and skin appendages. Finally, the last pattern is an example of risk factors typically associated with certain diseases in the long term, in this case possibly patients suffering from paralysis due to HCV or degenerative neurological diseases.

Although some of the patterns in both tables may seem obvious, we consider that they are still interesting as they not only confirm known risk factors, but also provide specific information about the time gap between antecedents and consequent. As mentioned above, the availability of more EHRs should allow our methodology to provide more specific patterns.

Albeit we are not including all the rules of the packs in this article, we would like to illustrate some phenomena with specific examples. For instance, the agreement in Table 3 showed that some results are not easy to interpret. Equation (9) shows an example of a patient-oriented pattern that was annotated differently by the experts in both *Correctness* and *Relevancy* aspects. One of the physicians found no relationship between antecedents and consequent, the second one mentioned that there was a clear relationship between them, while the third physician stated that both fever and lack of oxygen might cause neurological disturbances (for example convulsions). Eventually, by consensus, it was agreed that the rule was *Partially Correct (clinical facet)* and *Rel-Known* (Relevant and known).

9 $$\begin{array}{c} \text{Fever}\ \overset{5}{\Longrightarrow }\ \text {Shortness of breath/dyspnoea}\ \overset{5}{\Longrightarrow }\ {}\\ \text{Neurological symptom/complt. other}\end{array}$$

Equation (10) shows another example of an apriorilike pattern with disagreement among the experts: while one saw no relationship, another one thought that limitation in movement might lead to weight gain or, conversely, severe pain might lead to anorexia and weight loss. The third expert saw a debatable relationship. However, the consensus reached was that the pattern was not correct nor relevant. We consider Eqs. (9) and (10) as good examples of the intricacy and subjectivity of the evaluation process.

10 $$\begin{array}{c} \text{Pain general/multiple sites}\ \overset{16}{\Longrightarrow }\ \text{Pain} \\ \text{general/multiple sites}\ \overset{16}{\Longrightarrow }\ {} \\ \text{Endocrine/met./sympt/complt other} \end{array}$$

Equation (11) shows an example of a patient-oriented pattern that was annotated by the experts as *Rel-Known* and as *Par-Temporal* (Partially correct for the Temporal facet). They argued that the time elapsed among the medical events (in this case, all of the type symptom) was longer than expected, though it is indeed very often found indicating cases of complicated respiratory infections. This is an example of how our system can refute conventional knowledge and indicate that certain events may have a longer time frame to consider for prevention purposes.

11 $$\begin{array}{c} \text{Fever}\ \overset{79}{\Longrightarrow}\ \text{Pain general/multiple sites}\ \overset{86}{\Longrightarrow }\ {} \\ \text{Cough, Respiratory symptom/complaint other}\\ \overset{86}{\Longrightarrow }\ \text{Shortness of breath/dyspnoea} \end{array}$$

As noted above, some of the patterns were considered novel, i.e. *Relevant Unknown*, by some physicians in the individual ratings, although this assessment did not appear in the final consensus. For example, patient-oriented Eq. (12), which had a high value of the *rulesRespectConsequent* measure (20%), was considered a correct and *Rel-Known* pattern in the consensus. However, in the previous evaluations and during the consensus, two experts commented that they were hesitant about the correctness, but that if this relationship was considered correct, it would be novel to think that 20% of patients who have generalized pain and subsequently some neurological symptom would end up with a diagnosis of malignant neoplasm.

12 $$\begin{array}{c} \text{Pain general/multiple sites}\ \overset{15}{\Longrightarrow }\ \text{Neurological} \\ \text{symptom/complt. other}\ \overset{16}{\Longrightarrow }\ \text{Malignant} \\ \text{neoplasms} \end{array}$$

Finally, Eq. (13) shows another example of a possible evolution of symptoms that were categorized as *Relevant Unknown* by one physician. This patient-oriented pattern may capture a novel set of long-term risk factors typical of a patient with multiple pathologies that evolve in hypertension.

13 $$\begin{aligned} \begin{array}{c} {}\text {Cough, Respiratory symptom/complaint other}\\ {}\overset{83}{\Longrightarrow }\ \text {Paracetamol}\ \overset{87}{\Longrightarrow }\ \text {Cardiovascular}\\ {}\text {sympt/complt other, Entire limb}\ \overset{87}{\Longrightarrow }\ \text {Essential}\\ {}\text {(primary) hypertension} \end{array} \end{aligned}$$

In this section, we have just discussed some results. We are aware that our rules show low support, especially for long-term time gaps, as our data set only spans 6 years and these phenomena are more difficult to extract. Obtaining sequential patterns with high support from real-world healthcare data is generally difficult. For this reason, many researchers set a low minimum support. In our case, this situation is exacerbated by the limited dataset we analyzed, which consisted of the EHRs of only 320 patients with multimorbidity. To obtain more robust results, it is essential to have more extensive clinical data, both in terms of longer EHRs and a larger number of patients.

### Generalizability

In this work, we have focused on a very specific type of patient. However, it is certainly possible to use the same algorithms to mine temporal association rules from health records of different patient cohorts or with other medical conditions. In fact, the strength of our method lies in the possibility of extracting patterns from any patient population, provided a few considerations are taken into account:

- Dataset characteristics: The input dataset must contain electronic health records with the specific format, coding system and attributes to ensure compatibility with the algorithm. Therefore, depending on the specific patient population or medical conditions, performing additional data preprocessing steps may be required. This could involve handling missing values, normalizing or standardizing data, mapping different coding systems, or adapting the data representation to meet the requirements of the algorithm.
- Algorithm selection and parameter tuning: The same algorithm may require different parameter settings when applied to different datasets. It is important to carefully adjust the parameters to optimize the performance and quality of the temporal association rules discovered. Considerations include the minimum support threshold, the minimum confidence threshold, the number of time intervals (and the definition of each interval with a minimum and maximum time gap), and the appropriate approach (apriori or patient-oriented).
- Medical domain expertise: When applying our algorithm to different patient cohorts, it is crucial to involve experts in that specific domain. They can provide valuable insights and domain-specific knowledge to guide data preprocessing, algorithm selection and parameter tuning. Experts can also help validate the discovered temporal association rules and assess their clinical relevance and correctness.

By considering these aspects, our algorithm can be adapted to mine temporal association rules from health records of different patient populations or with other medical conditions. However, it is important to note that the results and interpretations may differ due to variations in the data and domain-specific factors. Therefore, thorough analysis, validation, and collaboration with domain experts are key to obtaining meaningful and actionable insights.

## Conclusions

A highly flexible temporal association rule mining algorithm, accompanied by a sound formulation for TARs, has been presented. The algorithm has been designed to be highly parameterizable, not only in terms of support and confidence measures but also in its ability to extract rules with different numbers of temporal steps (each one eventually with different time intervals [$t_1$, $t_2$]), attributes and levels of concept abstraction.

Using this algorithm, we have successfully extracted temporal association rules that help in the diagnosis and risk prediction of multimorbid patients. To achieve this, certain parameters were fixed, such as the use of codes as attributes, a fixed level of code generalization, and a minimum of 2 temporal steps in the rules. We experimented with different values for other parameters such as support, confidence, and time intervals. We aim to establish a strong basis for the future development of a robust rule extraction tool. Furthermore, due to the adaptability of our approach, it can be easily applied to the extraction of temporal association rules in various other domains.

In addition, our work proposes two different approaches, each with a unique rationale, allowing the choice of the one that best suits the medical dataset and the desired pattern application. The first approach is specifically designed for multimorbid patient datasets (though it might be tested with other patient types). On the other hand, the second approach is designed for conventional datasets and aims to extract rules that are common to patients. In this way, users can choose the approach that suits their specific needs and dataset characteristics.

We implemented a comprehensive evaluation process for the rules obtained. This process involved defining a set of evaluation criteria, agreed by three experts, covering both correctness and relevance. However, the practical evaluation proved to be challenging as it was heavily influenced by each expert’s experience and interpretation. To overcome this challenge, we conducted a subsequent consensus step and evaluated Fleiss’ Kappa agreement. The results demonstrated the subjectivity of the assessment process.

This evaluation process concluded that the implementations were able to retrieve correct and relevant rules. The apriori-like approach was found to be more accurate in terms of patterns, which may be due to the fact that the dataset used to retrieve the rules consisted of annotations from multimorbid patients with pre-selected characteristics. In some cases, especially in the long term, the rules obtained showed a relationship between pathologies that are highly prevalent. It should be noted that the insight behind such rules may not necessarily reflect a temporal cause-effect relationship, but rather a co-occurrence that signifies the patients’ comorbidity. Therefore, it would be beneficial to compare the patterns of multimorbid patients with those of general patients in order to identify multimorbid-specific temporal association rules, similar to what [11] did with diabetic-specific patterns.

It is important to note a final limitation related to the intrinsic code annotations in the dataset, particularly for *Sign or symptom* ICPC-3 codes. The majority of the selected rules contained codes that were too broad (e.g. *‘Respiratory symptom/complaint other’*), resulting in reduced relevance of the rules. This is a recurring problem in our clinical history corpus for two reasons. Firstly, the corpus is relatively small, which means that the most general phenomena tend to be more common. Secondly, primary care physicians are often overwhelmed and tend to assign a generic code because they do not have time to search for the most appropriate code for each case. This lack of time is a chronic problem in all primary care systems, and all physicians complain that EHRs would be more complete and specific if they had more time to enter patient information at each visit. This situation results in "noisy data", such as missing or incomplete data, inconsistent notation, irregular patient visits, and uncertainty about when healthcare events occurred versus when they were recorded. This problem is a drawback of our extraction system. However, an extension of our methodology could eventually provide suggestions and/or additional information to make this process of registering information more efficient. While intelligent diagnosis and treatment machines cannot replace human practitioners, they can assist them in making better clinical decisions.

The limitations of our dataset are related to both its size (in terms of both the number of available patients and the historical data per patient) and its nature (the aforementioned level of specificity of the annotations). Indeed, the size of a dataset can have an impact on the association rules mined from it. An important property that relates the size of the dataset to the association rules is the sparsity of the dataset. Sparsity refers to the proportion of rare itemsets in the dataset. In our case, the data set happens to be both small and sparse. This has meant that there are interesting phenomena that are not captured because the corresponding rules do not meet the minimum support. However, there are very general phenomena that have been apprehended by the methodology. Therefore we believe that, although larger datasets may lead to the discovery of more diverse and specific association rules, our current findings would still hold with greater reliability beyond our specific dataset. This is a hypothesis, it would be necessary to apply the methodology to an expanded dataset to assess the stability of the rules across different dataset sizes. If the rules obtained from the expanded dataset were consistent or similar to those obtained from the smaller dataset, this would suggest that the rules are robust and not solely influenced by the dataset size.

Despite these limitations, we have successfully extracted accurate and relevant patterns, particularly for the specific group of multimorbid patients. This methodology may be valuable for preventive medicine, as sequential temporal patterns can predict risk factors commonly associated with certain diseases. Therefore, the next step would be to integrate this set of patterns into the existing medical system so that the patterns could be used in the decision-making process. This integration involves representing the patients’ medical data in a format suitable for applying the temporal association rules. Then, to assess a patient’s risk factor for a disease, the following steps could be taken: (1) select the relevant temporal association rules that are likely to indicate the presence or increased risk of the specific disease of interest (this is indicated in the consequent of each rule), (2) apply the selected temporal association rules to the patient’s data and evaluate their applicability, i.e. check whether the antecedents of each rule match any relevant events in the patient’s medical history with the rule’s temporal constraints, and (3) for each applicable rule, determine the patient’s risk factor for the disease. If the rule has high support and confidence, it suggests a potential risk factor for the disease. The strength of the association and the temporal patterns captured by the rules can provide insight into the patient’s likelihood of developing the disease. Moreover, the average time gap provides crucial information about the term of these risk factors, which is vital in primary care.

At this stage, the only assessment of the quality of the extracted patterns we can provide is that of the experts. The patterns extracted by our algorithm align with clinical knowledge and can be easily understood by healthcare professionals. In our opinion, this demonstrates the potential of such patterns not only to confirm previously known associations but also to discover new relationships between factors and to better understand hypothesized relationships between such factors. We emphasize the importance of such transparent and explainable models in gaining the trust and acceptance of clinicians, enabling them to make informed decisions based on these patterns, thus empowering personalized preventive measures.

### Supplementary Information

**Additional file 1.**

## Data Availability

The data that support the findings of this study are available from Foundation University Institute for Primary Health Care Research Jordi Gol i Gurina (IDIAP JGol), but restrictions apply to the availability of these data, which were used under license for the current study, and so are not publicly available. Data are however available from the authors (AG and NC) upon reasonable request and with permission from IDIAP JGol.

## Abbreviations

EHR: Electronic health record
KDD: Knowledge discovery in databases
TAR: Temporal association rule
CAP: Primary Care Center (in Catalan)
ATC: Anatomical therapeutic chemical
ICD-10: International classification of diseases 10th revision
ICPC-3: International standard for Primary Care 3rd revision
SNOMED: Systematized Nomenclature of Medicine

## Footnotes

1 Throughout this work, we will use the terms patterns and rules interchangeably to refer to the concept of temporal association rules.

2 In addition, those rules containing sign or symptom with ICPC-3 code AS99 (‘Other specified general symptoms, complaints and abnormal findings’) and rules containing ICD-10 code Z76.8 (‘Persons encountering health services in other specified circumstances’) were filtered for being too general.

3 We represent them with equations in which the number appearing above the arrows represents the time constraint parameter (TC), i.e. the average time (in days) between temporal steps.
