# Supplementary material for: Acquisition of temporal patterns from electronic health records: an application to multimorbid patients
Source: BMC Med Inform Decis Mak. 2023 Sep 19;23:189. doi: 10.1186/s12911-023-02287-0 (PMC10510308; doi:10.1186/s12911-023-02287-0)
Supplement: Supplementary file 1 — Additional file 1. [file 12911_2023_2287_MOESM1_ESM.pdf]

Appendix

Description of nodes and relationships

Node properties: Body part (ParteCuerpo)

Supplementary table 1 Available data for ParteCuerpo (Body part)

Attribute	Description	Example/Possible Values
raw_text	Doctor's note.	ex: Abdominal
date	Date of the note.	ex: 20150102
code	Type of code and code.	ex: snomed:302553009
patient_id	Patient identifier.	ex: 345
id_node	Node identifier.	ex: 80054
text	Description based on the code.	ex: Abdomen
id	Node identifier within each patient. Follows temporal order.	ex: 755
dataset	Dataset where the data comes from	ex: idiap

Supplementary table 1 presents the properties of body parts. SNOMED codes are used to classify these instances. SNOMED (Systematized Nomenclature of Medicine, [1]) is a clinical reference terminology that enables healthcare professionals around the world to represent clinical information accurately and unambiguously, in a multilingual format. In this work we will not distinguish any kind of hierarchy in the SNOMED codes.

Node properties: Diagnosis (Diagnostico)

Supplementary table 2 Available data for Diagnostico (Diagnosis)

Attribute	Description	Example/Possible values
raw_text	Doctor's note	ex: hipertrofia de lobulo hepatico izquierdo
Date	Date of the note	ex: 20141231
cim10	More accurate cim10 code that can be related to diagnosis.	ex: K76.0
code	Type of code and code	ex: cim10:K76.0
patient_id	Patient identifier	ex: 345
id_node	Node identifier	ex: 81835
text	Description based on the code	x: Fatty (change of) liver, not elsewhere classified
id	Node identifier within each patient. Follows temporal order.	ex: 754
dataset	Dataset where the data comes from	ex: idiap

Supplementary table 2 shows the properties of diagnoses. ICD-10 codes are used to classify its instances. The ICD-10 code is a medical classification list created by the World Health Organization (WHO). It contains codes for diseases, signs and symptoms, abnormal findings, complaints, social circumstances and external causes of injury or diseases. However, we will only use the ICD-10 classification for diagnoses. The code has a non-trivial hierarchical structure, which can be found on the official page of the ICD-10 codes in Spanish (CIE-10 codes) [2]. Supplementary figure 1 intends to both show the complexity of the code and define the generalization levels.

The code is considered to have a non-trivial hierarchy due to the variation in the number of children of each node, the differing distance from the leaves to the root node, and the inconsistent labeling within each level. Supplementary figure 1 shows the largest path in bold, and we can see that the tree has a height of six levels.

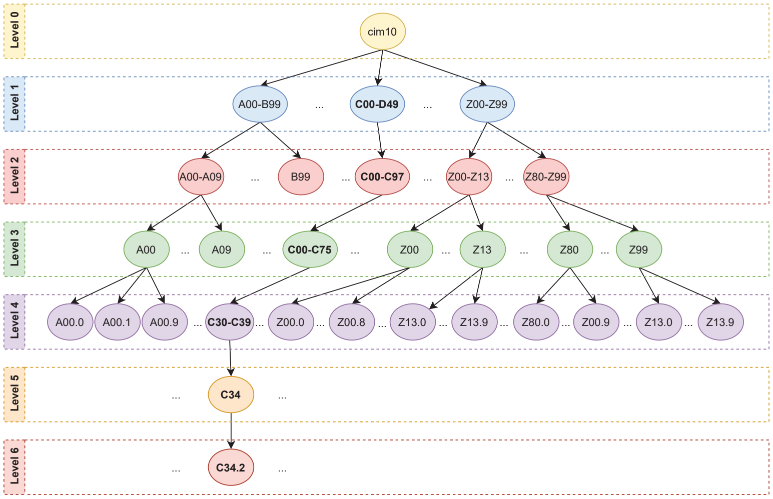

Supplementary figure 1 CIE-10 generalization tree summary

Supplementary table 3 Available data for *Farmaco* (Drug)

Attribute	Description	Example/Possible values
raw_text	Doctor's note	ex: Lexemma
date	Date of the note	ex: 20150217
code	Type of code and code	ex: atc7:D07AC14
patient_id	Patient identifier	ex: 345
id_node	Node identifier	ex: 79929
atc7	Most accurate ATC code that can be related to the drug	ex: D07AC14
text	Description based on the code	ex: methylprednisolone aceponate
id	Node identifier within each patient. Follows temporal order	ex: 756
dataset	Dataset where the data comes from	ex: idiap

Node properties: Drug (Farmaco)

Supplementary table 3 shows the properties of drugs. ATC codes are used to classify these instances. The Anatomical Therapeutic Chemical (ATC) Classification System [3] is a drug classification system controlled by the World Health Organization Collaborating Centre for Drug Statistics Methodology (WHOCC). The structure of the ATC codes is composed by different levels of hierarchy:

- The first level of the code indicates the anatomical main group. It consists of one letter.
- The second level of the code indicates the therapeutic subgroup. It consists of two digits.
- The third level of the code indicates the therapeutic/pharmacological subgroup. It consists of one letter.
- The fourth level of the code indicates the chemical/therapeutic/pharmacological subgroup. It consists of one letter.
- The fifth level of the code indicates the chemical substance. It consists of two digits.

Supplementary figure 2 shows a summary of the generalization tree of the ATC7 code as well as our definition of the different level numbers. We can observe that although the maximum tree height is five, not all nodes have the same number of

children, so the distance from all leaves to the root node is not the same. However, we can see that all codes belonging to the same level follow the same structure.

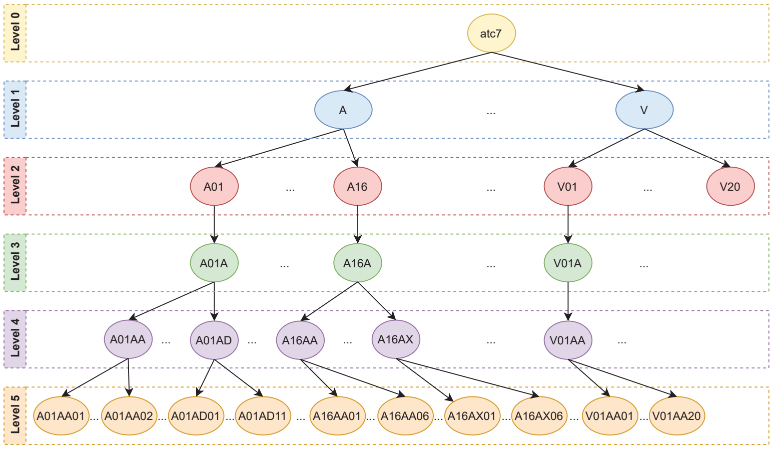

Supplementary figure 2 ATC7 generalization tree summary

Node properties: Sign or symptom (SignoSintoma)

Supplementary table 4 Available data for *SignoSintoma* (Sign or symptom)

Attribute	Description	Example/Possible Values
raw_text	Doctor's note	ex: odinofagia
date	Date of the note	ex: 20150217
code	Type of code and code	ex: ciap2:D21
patient_id	Patient identifier	ex: 345
id_node	Node identifier	ex: 80635
text	Description based on the code	ex: Swallowing problem
id	Node identifier within each patient. Follows temporal order.	ex: 761
dataset	Dataset where the data comes from	ex: idiap

Supplementary table 4 depicts the properties of signs or symptoms. CIAP-2 codes are used to classify its instances. A CIAP-2 code (Spanish for International Classification of Primary Care, ICPC-3) is a three digit code where:

- The first character is a letter that represents an organ or system. There are 17 possible values.
- The second and third characters are digits that include three groups of components (complaints, processes of care, and health problems).

Supplementary figure 3 features a summary of the generalization tree of the CIAP-2 code and the different level numbers. The image is simplified, but it doesn't mean that all the nodes have the same number of children. Unlike ATC7, CIAP-2 codes have a uniform distance between each leaf and the root node.

Relationships

There are six types of relationships: *before*, *causality_of*, *coOccur*, *cotreated_with* (treated at the same time than), *located_in*, and *substituted_by*.

coOccur relationship connects all pairs of nodes that belong to the same visit. We define a visit as all the healthcare instances that have the same *patient_id* and

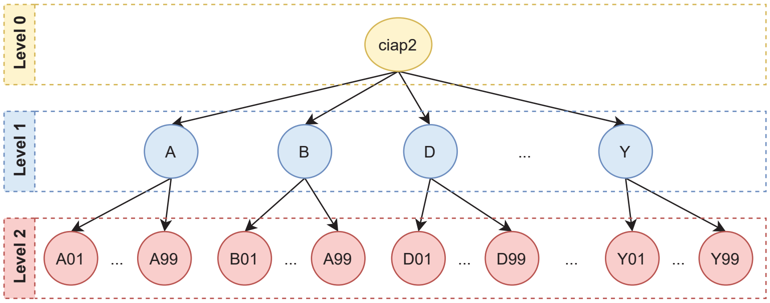

Supplementary figure 3 CIAP-2 generalization tree summary

date properties. That is, we assume that patients can only attend one visit per day. *before* relationship connects all pairs of nodes that belong to two consecutive visits. *coOccur* and *before* connections can be observed in Supplementary figure 4, where four visits for a certain patient are shown: day 20161109 (in the red circle), day 20161110 (in the orange circle), day 20161111 (in the yellow circle) and day 20161112 (in the brown circle).

The other relationships *causality_of*, *cotreated_with*, *located_in* and *substituted_by* provide additional information between nodes, independently of the temporal relation.

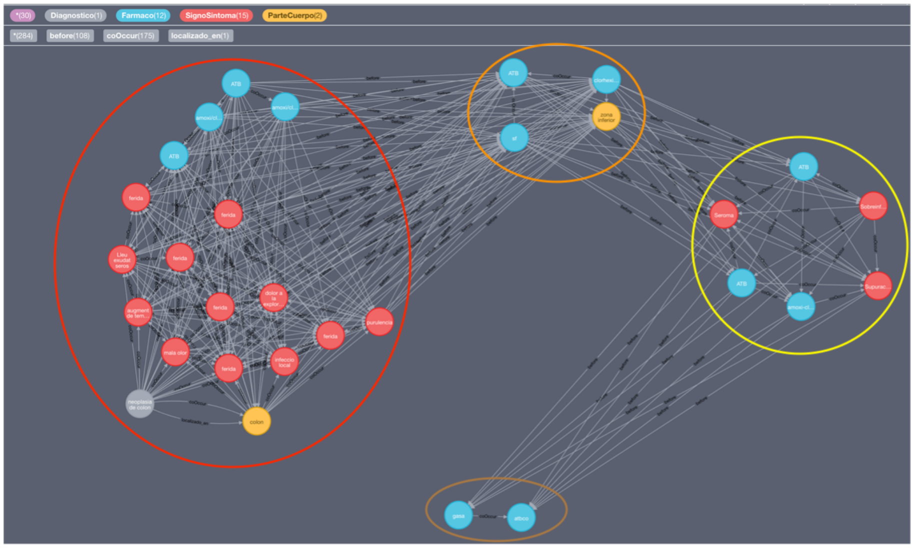

Supplementary figure 4 Healthcare instances from patient 32, days 20161109, 20161110, 20161111 and 20161112

References

1. SCT. Systematized Nomenclature of Medicin; 2022. Available from: `https://www.snomed.org/`.
2. World Health Organization. Clasificación Internacional de Enfermedades; 2022. Available from: `https://icdcode.info/espanol/cie-10/codigos.html`.
3. World Health Organization. Anatomical Therapeutic Chemical (ATC) Classification; 2022. Available from: `https://www.who.int/tools/atc-ddd-toolkit/atc-classification`.
